# Broadening horizons: research on ferroptosis in lung cancer and its potential therapeutic targets

**DOI:** 10.3389/fimmu.2025.1542844

**Published:** 2025-01-23

**Authors:** Guangpeng Gao, Xindi Zhang

**Affiliations:** Department of Pulmonary Disease (Department of Respiratory and Critical Care Medicine), Gansu Provincial Hospital of Traditional Chinese Medicine, Lanzhou, China

**Keywords:** lung cancer, ferroptosis, cell death mechanisms, tumor microenvironment, therapeutic strategies, molecular pathways

## Abstract

Ferroptosis is a novel form of cell death distinct from traditional mechanisms, characterized by the accumulation of iron ions and the production of lipid peroxides. It not only affects the survival of tumor cells but is also closely linked to changes in the tumor microenvironment. Lung cancer is one of the leading malignancies worldwide in terms of incidence and mortality, and its complex biological mechanisms and resistance make treatment challenging. Recent studies have shown that ferroptosis plays a key role in the onset and progression of lung cancer, with its intricate regulatory mechanisms influencing tumor development and response to therapy. As research into ferroptosis deepens, related molecular pathways, such as glutamate metabolism, iron metabolism, and antioxidant defense, have been gradually revealed. However, in clinical practice, ferroptosis-based therapeutic strategies for lung cancer are still in their early stages. Challenges remain, including the incomplete understanding of the specific mechanisms of ferroptosis, insufficient research on related regulatory factors, and limited insight into the interactions within the tumor microenvironment. Therefore, effective modulation of ferroptosis to enhance lung cancer treatment remains an urgent issue. This review summarizes the biological mechanisms of ferroptosis, analyzes the regulatory factors of ferroptosis in lung cancer cells and their interaction with the tumor microenvironment, and further explores potential therapeutic strategies targeting ferroptosis. By synthesizing the latest research, this paper aims to provide new perspectives and directions for lung cancer treatment, with the goal of advancing clinical applications.

## 1 Introduction

Lung cancer is one of the most common cancers worldwide and has an extremely high mortality rate (1). According to global cancer statistics, the annual death toll from lung cancer has surpassed 2 million, making it one of the leading causes of cancer-related deaths (2). In some regions, the incidence and mortality rates of lung cancer continue to rise year by year, posing a significant public health challenge (3–6). The high mortality rate of lung cancer is primarily attributed to factors such as the lack of obvious early symptoms, late diagnosis, and treatment difficulties. Additionally, smoking, air pollution, occupational exposure, and other factors are considered major risk factors for lung cancer, with their impact varying significantly across different regions (7).

Traditional treatment methods for lung cancer mainly include surgery, radiotherapy, and chemotherapy. However, these approaches have significant limitations when treating advanced-stage lung cancer, especially small cell lung cancer (SCLC) and non-small cell lung cancer (NSCLC) (8). Surgical treatment is effective for early-stage cases, but in patients with locally advanced or metastatic lung cancer, chemotherapy and radiotherapy often show limited effectiveness and are accompanied by high toxic side effects (9, 10). Moreover, due to tumor heterogeneity and drug resistance, many patients still face the risk of recurrence and metastasis after receiving conventional treatments (11). Despite advancements in early screening and treatment methods for lung cancer in recent years, the prognosis remains poor, with a five-year survival rate of less than 20% (12). Therefore, there is an urgent need to explore new treatment strategies to improve the survival rate and quality of life for lung cancer patients. A comparison of the advantages and disadvantages of various lung cancer treatment methods is shown in **Table 1**:

**Table 1:** The advantages and disadvantages of current treatments for lung cancer.

Therapies	Advantages	Disadvantages
Surgical excision	1. Directly removing the tumor may lead to a cure. 2. Significant efficacy for early-stage lung cancer.	1. The trauma is significant, and the recovery time is long. 2. It may cause complications, such as infection and bleeding. 3. The range of resection is limited and is suitable for some early-stage lung cancers. 4. Some patients are not suitable for surgery due to their physical condition.
Radiation therapy	1. Good control effect on local tumors. 2. Suitable for some patients who cannot undergo surgery. 3. Combined with chemotherapy can improve efficacy.	1. May cause side effects such as radioactive pneumonia and esophagitis. 2. Long-term radiotherapy may lead to skin damage, fatigue, etc. 3. Radiotherapy equipment and technology have high requirements.
Chemotherapy	1. Inhibitory effect on tumor cells throughout the body. 2. Suitable for advanced lung cancer and adjuvant therapy after surgery. 3. Combined with radiotherapy can enhance efficacy.	1. The side effects are significant, such as nausea, vomiting, and hair loss. 2. The issue of drug resistance may affect efficacy. 3. Chemotherapy drugs are expensive, placing a heavy financial burden on patients.
Targeted therapy	1. Highly targeted with relatively minor side effects. 2. Long-lasting efficacy and higher quality of life for patients. 3. Combining with chemotherapy can enhance efficacy.	1. Only applicable to lung cancer patients with specific gene mutations. 2. The drug is expensive, placing a heavy financial burden on patients. 3. There may be issues with drug resistance.
Immunotherapy	1. Significant efficacy for patients with advanced lung cancer. 2. Significantly improved patients’ survival rates and quality of life. 3. Combined with chemotherapy and radiotherapy, it can enhance efficacy.	1. Possible side effects such as autoimmune reactions may occur. 2. Treatment costs are high, placing a heavy financial burden on patients. 3. Efficacy and safety vary due to individual differences.

Iron plays a crucial role in cellular metabolism, as it is involved not only in oxygen transport and energy metabolism, but also in DNA synthesis and repair (13). However, excessive iron accumulation can lead to an increase in reactive oxygen species (ROS) within cells, triggering oxidative stress that damages cellular DNA and other macromolecules, thereby promoting tumor initiation and progression (14, 15). In recent years, increasing evidence has shown that dysregulation of iron metabolism is closely associated with the development of various cancers, including lung cancer (16). Iron is not only a key element required for tumor cell growth but also a critical regulator of cell death. In particular, in lung cancer, abnormal iron metabolism may represent an important feature of the tumor microenvironment, influencing tumor cell growth and metastasis.

In this context, ferroptosis, a newly discovered form of programmed cell death, has gradually gained attention from researchers. Ferroptosis is a form of cell death driven by the accumulation of iron-dependent lipid peroxides, and it differs from traditional forms of cell death such as apoptosis and necrosis (17, 18). For instance, cells undergoing ferroptosis typically exhibit membrane rupture and organelle swelling, changes not typically observed in apoptosis. Ferroptosis was initially identified in tumor research, but more recent studies have indicated that it also plays a significant role in various neurodegenerative diseases, such as Alzheimer’s disease and Parkinson’s disease, as well as other pathological conditions. The mechanism of ferroptosis involves multiple biological processes, including iron metabolism, lipid metabolism, and antioxidant responses (19, 20). Importantly, ferroptosis plays a critical role in the tumor microenvironment, and inducing ferroptosis may offer novel therapeutic strategies for cancer treatment (21). By modulating iron metabolism and lipid peroxidation, ferroptosis can be induced in tumor cells, thereby inhibiting tumor growth and metastasis, and overcoming resistance to traditional therapies (22, 23).

In lung cancer, the expression profiles of GPX4 (glutathione peroxidase 4) and SLC7A11 (solute carrier family 7 member 11) are closely associated with patient prognosis. Studies have shown that high expression of GPX4 in lung adenocarcinoma (LUAD) is linked to tumor aggressiveness and drug resistance, with its expression level negatively correlated with patient survival. Similarly, high expression of SLC7A11 is considered a marker of poor prognosis in lung cancer patients. Specifically, overexpression of SLC7A11 is associated with tumor stage, lymph node metastasis, and chemotherapy resistance. Furthermore, the expression patterns of ferroptosis-related genes can serve as biomarkers to predict survival and therapeutic response in lung cancer patients. For example, one study developed a prognostic model based on ferroptosis-related genes using RNA-seq data from NSCLC patients, revealing that patients in the high-risk group had significantly lower overall survival compared to those in the low-risk group (24).

These findings indicate that ferroptosis-related genes not only play a pivotal role in the initiation and progression of lung cancer but also have potential clinical applications, providing a foundation for the development of personalized treatment strategies. Therefore, exploring the role of ferroptosis in lung cancer not only aids in understanding the biological characteristics of the disease but may also provide important insights for the development of novel therapeutic approaches.

## 2 The definition and mechanism of ferroptosis

### 2.1 Biological characteristics of ferroptosis

Ferroptosis is a novel form of programmed cell death that differs significantly from traditional mechanisms of cell death in its biological characteristics. The hallmark of ferroptosis is the accumulation of iron-dependent lipid peroxides, leading to cell membrane rupture and cell death. Additionally, the morphological features of ferroptosis cells differ from other forms of cell death, typically exhibiting cell shrinkage, disruption of cell membrane integrity, and the formation of intracellular lipid vesicles (25, 26). Ferroptosis is closely associated with excessive intracellular iron accumulation and the generation of ROS, which together lead to lipid peroxidation and cellular damage. Research has shown that ferroptosis not only plays a crucial role in cancer cells but is also closely linked to the pathogenesis of various diseases, including neurodegenerative diseases, cardiovascular diseases, and kidney disorders (27–32).

**Figure 1:**
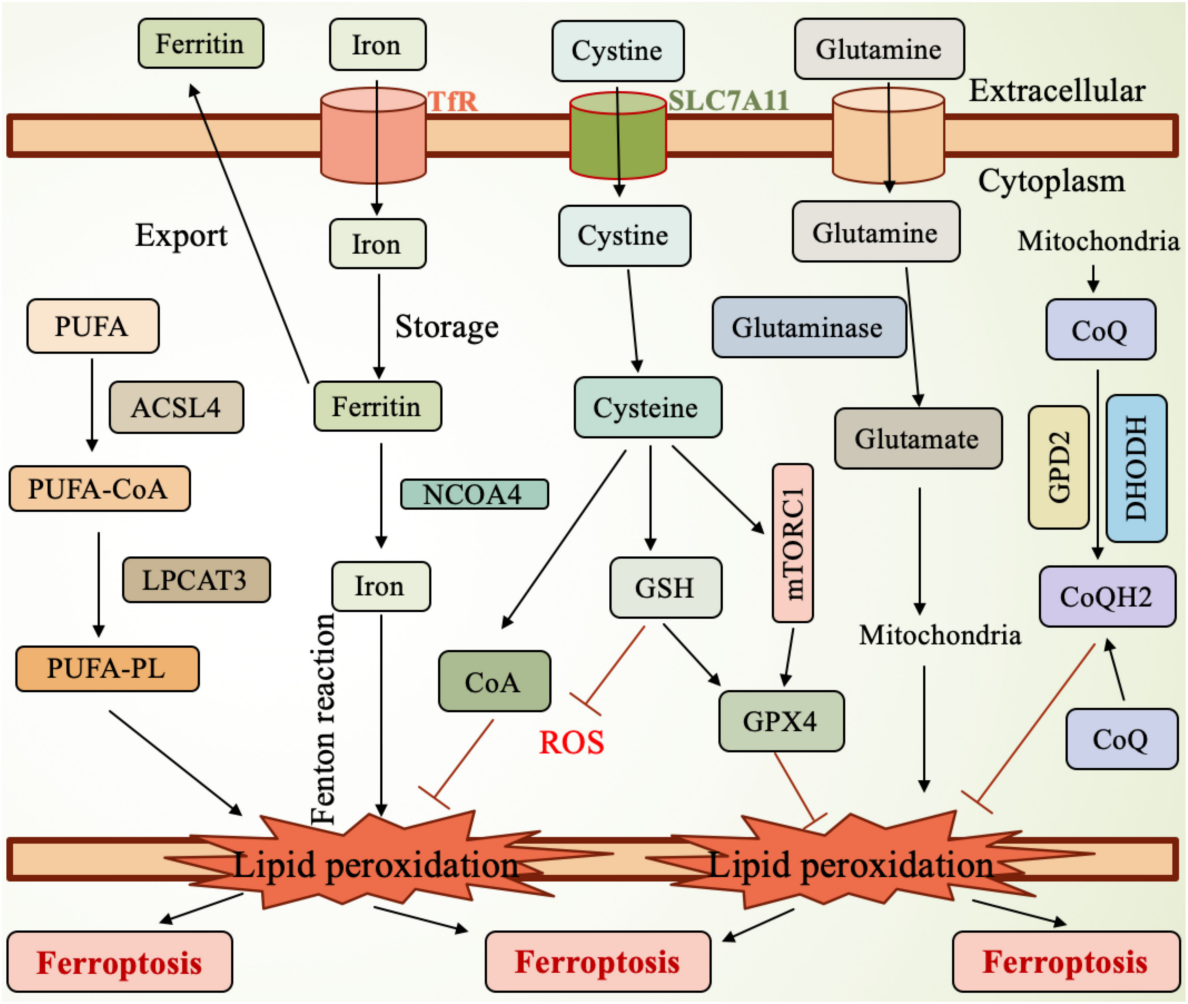
Mechanism of ferroptosis. SLC7A11, the cystine transporter solute carrier family member 11; GSH, glutathione; ROS, reactive oxygen species; GPX4, glutathione peroxidase 4; mTORC1, mammalian target of rapamycin complex 1; GPD2, glycerol-3-phosphate dehydrogenase 2; DHODH, dihydroorotate dehydrogenase; CoQH2, ubiquinol; TfR, transferrin receptor; NCOA4, nuclear receptor coactivator 4; PUFA, polyunsaturated fatty acid; ACSL4, Acyl-CoA synthetase long-chain family member 4; LPCAT3, lysophosphatidylcholine acyltransferase 3; PUFA-PL, PUFA-containing phospholipids.

The regulation of ferroptosis involves multiple molecular pathways and signaling cascades. Key molecules, such as GPX4 and SLC7A11, are central to ferroptosis regulation (**Figure 1**). GPX4 plays a critical protective role during ferroptosis, and its loss of function increases the cell’s susceptibility to ferroptosis. By catalyzing the reduction of lipid peroxides, GPX4 protects cells from oxidative damage and is therefore considered an inhibitor of ferroptosis. On the other hand, SLC7A11 is responsible for the uptake of cysteine, which is essential for glutathione synthesis, thereby enhancing the cell’s resistance to oxidative stress and suppressing ferroptosis.

In lung cancer, abnormal expression of ferroptosis-related molecules is considered closely linked to tumor initiation and progression. The expression levels of GPX4 and SLC7A11 are significantly upregulated in lung cancer cells, and this upregulation correlates with the malignancy and prognosis of the tumor, making them potential therapeutic targets (33, 34). Studies have shown that high expression of SLC7A11 is closely associated with the proliferation and survival of lung cancer cells, while the expression of GPX4 is linked to chemo resistance (35). Additionally, other ferroptosis-related molecules, such as NCOA4 (nuclear receptor coactivator 4) and TXNIP (thioredoxin-interacting protein), also play important roles in ferroptosis in lung cancer (36). Therefore, targeted therapeutic strategies aimed at these molecules may offer new treatment options for lung cancer patients, particularly in the management of drug-resistant tumors (37). Furthermore, ferroptosis inducers and inhibitors affect its occurrence through different signaling pathways, further highlighting its complex biological characteristics (38).

The study of ferroptosis provides new insights into the mechanisms of cell death and its role in disease, especially in the development of therapeutic strategies for cancer and neurodegenerative disorders. Thus, a deeper understanding of the biological features and underlying mechanisms of ferroptosis is crucial for developing targeted treatments for related diseases.

### 2.2 Molecular mechanisms of ferroptosis

The molecular mechanisms of ferroptosis primarily involve iron metabolism, lipid peroxidation, and related signaling pathways (**Figure 1**). Excessive iron accumulation in cells can generate ROS through the Fenton reaction, leading to lipid peroxidation (39). Lipid peroxidation is one of the core events in ferroptosis, as excessive ROS generation oxidizes polyunsaturated fatty acids (PUFAs) in cell membranes, forming lipid peroxides that further trigger cell death (40–42). Studies have shown that ferroptosis is closely related to the depletion of glutathione (GSH), an important antioxidant. A lack of GSH reduces the cell’s ability to resist oxidative stress, thereby promoting ferroptosis (43, 44). Furthermore, GSH depletion is often associated with iron overload, a state that predisposes cells to ferroptosis, which has been validated in various diseases, such as acute kidney injury and neurodegenerative diseases (45).

GPX4 is a critical regulator in preventing ferroptosis, as it inhibits ferroptosis by catalyzing the reduction of lipid peroxides (**Figure 1**). Therefore, dysfunction of GPX4 leads to increased ferroptosis, while iron deficiency or failure of related antioxidant systems can exacerbate this process (46, 47). Additionally, ferroptosis is also associated with the activation of several signaling pathways, with the Nrf2 pathway playing a key role in regulating the cellular antioxidant capacity (48). Recent research has increasingly highlighted the role of non-coding RNAs (such as miRNAs and lncRNAs) in the regulation of ferroptosis. These molecules can influence the process of ferroptosis by modulating the expression of related genes (49–52). In summary, the molecular mechanisms of ferroptosis are complex and involve interactions among multiple biological pathways (**Figure 1**). By modulating lipid metabolism and glutathione levels, new strategies for treating related diseases may emerge. Research in this field is continuously advancing, providing a theoretical foundation for clinical applications.

### 2.3 The relationship between iron metabolism and oxidative stress

Oxidative stress refers to the imbalance in the cellular environment caused by excessive ROS production and insufficient antioxidant capacity, leading to cell damage and death (**Figure 1**). There is a close interplay between iron metabolism and oxidative stress. Iron, an essential trace element, participates in various biochemical reactions within cells, but excess iron can promote ROS generation, triggering oxidative stress (53, 54). Oxidative stress not only directly damages cell membranes and organelles but also activates several signaling pathways that further exacerbate iron accumulation and cellular damage. In diseases affecting organs such as the kidneys and liver, abnormal iron metabolism is closely related to oxidative stress, and together they promote cellular damage and dysfunction (27, 55). For instance, in conditions of iron overload, increased ROS within cells can further amplify iron accumulation, creating a vicious cycle that ultimately leads to ferroptosis (56, 57). Moreover, oxidative stress can also influence the expression of proteins involved in iron metabolism, creating a feedback loop that accelerates cell death under the combined pressures of oxidative stress and iron metabolism imbalance (58). Understanding the relationship between iron metabolism and oxidative stress is crucial for developing new therapeutic strategies to address related diseases (**Figure 1**).

### 2.4 The role of ferroptosis in tumor immunity

Ferroptosis plays a complex role in tumor immunity. Recent studies have shown that ferroptosis not only serves as a form of tumor cell death but also influences immune cell functions within the tumor microenvironment. Ferroptosis can induce immunogenic cell death, and the released cellular contents (such as damage-associated molecular patterns, or DAMPs) can activate dendritic cells, thereby enhancing anti-tumor immune responses (59). However, inhibition of ferroptosis may allow tumor cells to escape immune surveillance, reducing the effectiveness of immunotherapy (60). Additionally, the combination of ferroptosis and immune checkpoint inhibition (ICI) therapy has shown potential synergistic effects, possibly improving the efficacy of cancer treatments (61). Studies suggest that ferroptosis can enhance the effects of immunotherapy by inducing immunogenic death of tumor cells and activating anti-tumor immune responses. However, the oxidative byproducts released during ferroptosis may also inhibit immune cells in the tumor microenvironment, leading to immune tolerance, which in turn affects the outcome of immunotherapy (62). For example, tumor-associated macrophages can secrete certain metabolites that promote tumor cell resistance to ferroptosis, further influencing tumor progression and the effectiveness of immunotherapy (63). Therefore, in-depth research into the role of ferroptosis in tumor immunity not only helps us understand the mechanisms of immune escape in tumors but may also provide a theoretical foundation for developing new immunotherapeutic strategies.

## 3 The role of ferroptosis in lung cancer

### 3.1 Abnormal iron metabolism in lung cancer cells

Iron metabolism in lung cancer cells is notably dysregulated, and this dysregulation is closely linked to the occurrence of ferroptosis. Studies show that lung cancer cells typically exhibit excessive iron accumulation, which leads to an increase in ROS, triggering lipid peroxidation and ultimately resulting in ferroptosis. In lung cancer cells, the disruption of iron metabolism is characterized by upregulation of iron transport proteins such as transferrin (Tf) and hepcidin, while the expression of ferroportin, an iron export protein, is reduced (**Figure 1**). These alterations affect the cell’s ability to uptake and store iron, thus promoting tumorigenesis and progression (64–66). Additionally, some studies have found abnormal expression of ferroptosis-related genes in lung cancer patients, suggesting that the alteration of iron metabolism could serve as a new therapeutic target for lung cancer treatment (67, 68). Therefore, regulating iron metabolism and enhancing ferroptosis to inhibit tumor growth may represent a novel therapeutic strategy for lung cancer.

Iron metabolism plays a crucial role in the onset and progression of lung cancer. Research indicates that dysregulated iron metabolism not only promotes tumor growth but is also closely linked to metastasis and drug resistance. The cellular state of iron (such as ferrous iron Fe^2+^ and ferric iron Fe^3+^) exerts different effects on the growth and survival of lung cancer cells. For instance, studies have shown that the accumulation of ferrous iron inhibits the proliferation of lung cancer cells, whereas ferric iron has little impact on cell growth (69). Moreover, cancer-associated fibroblasts (CAFs) regulate the iron metabolism of lung cancer cells by secreting exosomes, which in turn affect the occurrence of ferroptosis. This process involves the interaction of long non-coding RNAs (such as ROR1-AS1) with genes related to iron metabolism. Disruption of iron metabolism not only accelerates lung cancer progression but may also contribute to treatment resistance. Therefore, further investigation into the interplay between iron metabolism and lung cancer progression could provide new strategies and targets for therapy. Overall, the interaction between ferroptosis and iron metabolism offers new insights into the onset and development of lung cancer, highlighting the importance of iron as a potential therapeutic target.

### 3.2 The relationship between ferroptosis and lung cancer cell proliferation

Ferroptosis plays a dual role in lung cancer cell proliferation. On one hand, the induction of ferroptosis can suppress lung cancer cell proliferation. Research suggests that by regulating iron metabolism and inducing ferroptosis, the growth and spread of lung cancer cells can be effectively inhibited. For example, ubiquitin-specific protease 35 (USP35), a deubiquitinase, when overexpressed in lung cancer cells, stabilizes ferroportin and prevents ferroptosis, thus promoting cell proliferation (70). In contrast, inhibiting USP35 expression leads to an increase in ferroptosis and a suppression of lung cancer cell proliferation. On the other hand, inhibition of ferroptosis may accelerate the proliferation of lung cancer cells, which is closely associated with the malignancy and chemotherapy resistance of the tumor (71). Additionally, long non-coding RNAs (lncRNAs) also play a crucial role in regulating ferroptosis. For example, the upregulation of LUCAT1 is associated with increased lung cancer cell proliferation, while its inhibition promotes ferroptosis. Therefore, modulating ferroptosis pathways may offer new therapeutic approaches for lung cancer, particularly in overcoming chemotherapy resistance, by inducing ferroptosis to enhance tumor cell sensitivity to treatment (71, 72).

### 3.3 The role of ferroptosis in lung cancer metastasis

Ferroptosis also plays an important role in the metastasis of lung cancer. Studies have found that ferroptosis can promote lung cancer metastasis by modulating the tumor microenvironment and affecting immune cell function. For example, the occurrence of ferroptosis may lead to the release of pro-inflammatory cytokines from tumor cells, which alters the tumor microenvironment and influences the metastatic capacity of the cancer (19). Some studies suggest that after ferroptosis, immune cells such as macrophages and neutrophils in the tumor microenvironment become activated and release pro-inflammatory factors, further promoting tumor growth and metastasis (71, 73). Moreover, ferroptosis may influence the epithelial-mesenchymal transition (EMT) process in tumor cells, thus enhancing the invasiveness and metastatic potential of lung cancer cells (74). Research indicates that inhibiting ferroptosis could aid in suppressing lung cancer cell metastasis. For instance, CAFs secrete lncRNAs (such as ROR1-AS1) in exosomes to inhibit ferroptosis in lung cancer cells, thereby promoting metastasis (75). Furthermore, the dysregulation of iron metabolism is closely associated with the metastatic ability of tumors. Iron accumulation not only promotes tumor cell proliferation but also enhances oxidative stress and induces inflammation, which can further promote metastasis (76). Therefore, therapeutic strategies targeting ferroptosis may help inhibit lung cancer metastasis and improve patient prognosis. By modulating iron metabolism and inducing ferroptosis, new anti-metastasis therapies could be developed, offering better treatment options for lung cancer patients.

## 4 Regulatory factors of ferroptosis

### 4.1 Lipid peroxides and their metabolic enzymes

Ferroptosis is an iron-dependent form of cell death, characterized by the accumulation of lipid peroxides in the cell membrane. The generation of lipid peroxides is closely linked to intracellular iron levels, with excessive iron accumulation driving the production of ROS through the Fenton reaction, which, in turn, exacerbates lipid peroxidation (**Figure 1**). Studies have shown that the sensitivity to ferroptosis is regulated by various lipid metabolic enzymes, such as long-chain acyl-CoA synthetase 4 (ACSL4) and lipoxygenases (LOX) (**Figure 1**). These enzymes play critical roles at different stages of lipid peroxidation, influencing the cell’s response to ferroptosis. For example, an increase in ACSL4 expression promotes the accumulation of polyunsaturated fatty acids (PUFAs), which enhances the sensitivity of cells to ferroptosis (**Figure 1**). The regulation of these metabolic enzymes not only affects cell survival but is also closely associated with the development of various diseases, including cancer and neurodegenerative disorders (77, 78). Furthermore, the presence of PUFAs in the cell membrane significantly impacts the occurrence of ferroptosis, as these PUFAs are highly susceptible to peroxidation, leading to membrane damage and cell death (39). Therefore, regulating the activity of lipid metabolic enzymes and maintaining the balance of lipid components are crucial strategies for modulating ferroptosis, providing new insights and targets for the treatment of related diseases (79–81).

### 4.2 The role of antioxidants in ferroptosis

Antioxidants play a crucial role in the regulation of ferroptosis. The occurrence of ferroptosis is often accompanied by the dysregulation of the intracellular antioxidant system, particularly the loss of function of GPX4, which significantly reduces the cell’s ability to defend against lipid peroxidation (**Figure 1**). For example, in the tumor microenvironment, the imbalance of antioxidants can lead to the accumulation of iron and the increase of lipid peroxides, thereby inducing ferroptosis. Therefore, the role of antioxidants is highly dependent on the cellular environment and specific physiological and pathological conditions (82, 83). Research has shown that antioxidants such as vitamin E and other natural compounds can effectively inhibit the occurrence of ferroptosis by enhancing the cell’s antioxidant capacity and mitigating the impact of lipid peroxidation. Additionally, nuclear factor erythroid 2-related factor 2 (Nrf2), a key transcription factor, can regulate the expression of antioxidant genes within the cell, thereby influencing the sensitivity to ferroptosis. Thus, utilizing antioxidants to boost the cell’s antioxidant capacity may become a novel strategy for treating diseases associated with ferroptosis (84–86).

### 4.3 The impact of tumor microenvironment on ferroptosis

The tumor microenvironment is one of the key regulators of ferroptosis. The metabolic characteristics of tumor cells in this environment, such as hypoxia, acidity, and nutrient deprivation, can influence iron metabolism and the oxidative state of lipids, thereby modulating the sensitivity to ferroptosis (87). Studies have shown that the interactions between immune cells and tumor cells in the tumor microenvironment may affect tumor progression and metastasis by regulating ferroptosis pathways. For example, certain immune cells in the tumor microenvironment might promote ferroptosis in tumor cells or help them resist ferroptosis through the release of cytokines and metabolic byproducts. Moreover, tumor cells’ ability to adapt to ferroptosis may contribute to resistance to anticancer therapies. Additionally, the high iron concentration and oxidative stress present in the tumor microenvironment can also promote the occurrence of ferroptosis, a mechanism that plays a crucial role in tumor progression and metastasis. Therefore, further research into the influence of the tumor microenvironment on ferroptosis will not only help to understand tumor biology but also provide a basis for the development of new therapeutic strategies (81, 88, 89).

## 5 Interaction between ferroptosis and other forms of cell death

### 5.1 Interaction between ferroptosis and apoptosis

Ferroptosis is a newly identified form of programmed cell death, primarily driven by iron-dependent lipid peroxidation, leading to cellular death. In contrast, apoptosis is executed through intracellular signaling pathways, particularly via the activation of caspases. Therefore, ferroptosis differs significantly from classical apoptosis. Studies have shown a complex interplay between ferroptosis and apoptosis. On one hand, in certain contexts, ferroptosis can induce apoptosis by promoting the generation of ROS within the cell. For instance, lncRNA LINC00618 has been found to promote both apoptosis and ferroptosis in leukemia cells, suggesting an interaction between these two cell death mechanisms (90). On the other hand, ferroptosis may inhibit apoptosis. For example, in some cancer cells, the activation of ferroptosis may suppress apoptotic pathways, thereby affecting tumor growth and metastasis (91). Furthermore, in some cases, ferroptosis may occur prior to apoptosis, resulting in cell death before apoptotic signals emerge (92). Thus, a deeper understanding of the interaction between ferroptosis and apoptosis not only helps unravel the complexity of cell death mechanisms but may also provide new avenues for cancer therapy (93).

### 5.2 Relationship between ferroptosis and necroptosis

Necroptosis is a regulated form of necrosis, typically triggered by intracellular death receptor signaling, and shares morphological and biochemical features with ferroptosis. Both processes involve rupture of the cell membrane and the release of cellular contents, triggering inflammatory responses (94). Research has shown that ferroptosis and necroptosis interact in certain contexts. For instance, iron overload can promote necroptosis, and the activation of necroptosis can exacerbate ferroptosis by promoting iron release (95). Excessive iron accumulation can not only induce ferroptosis but may also promote necroptotic cell death by affecting mitochondrial function and the integrity of the cell membrane (96). Additionally, certain inhibitors, such as KW-2449 and Necrostatin-1, have been found to suppress both cell death pathways simultaneously, suggesting that they may share common regulatory mechanisms. In pathological conditions like ischemia-reperfusion injury, ferroptosis and necroptosis work together in the cellular damage process, regulating the type and extent of cell death (97). Therefore, combined intervention targeting these two forms of cell death may offer new strategies for treating related diseases.

### 5.3 Interaction between ferroptosis and autophagy

**Figure 2:**
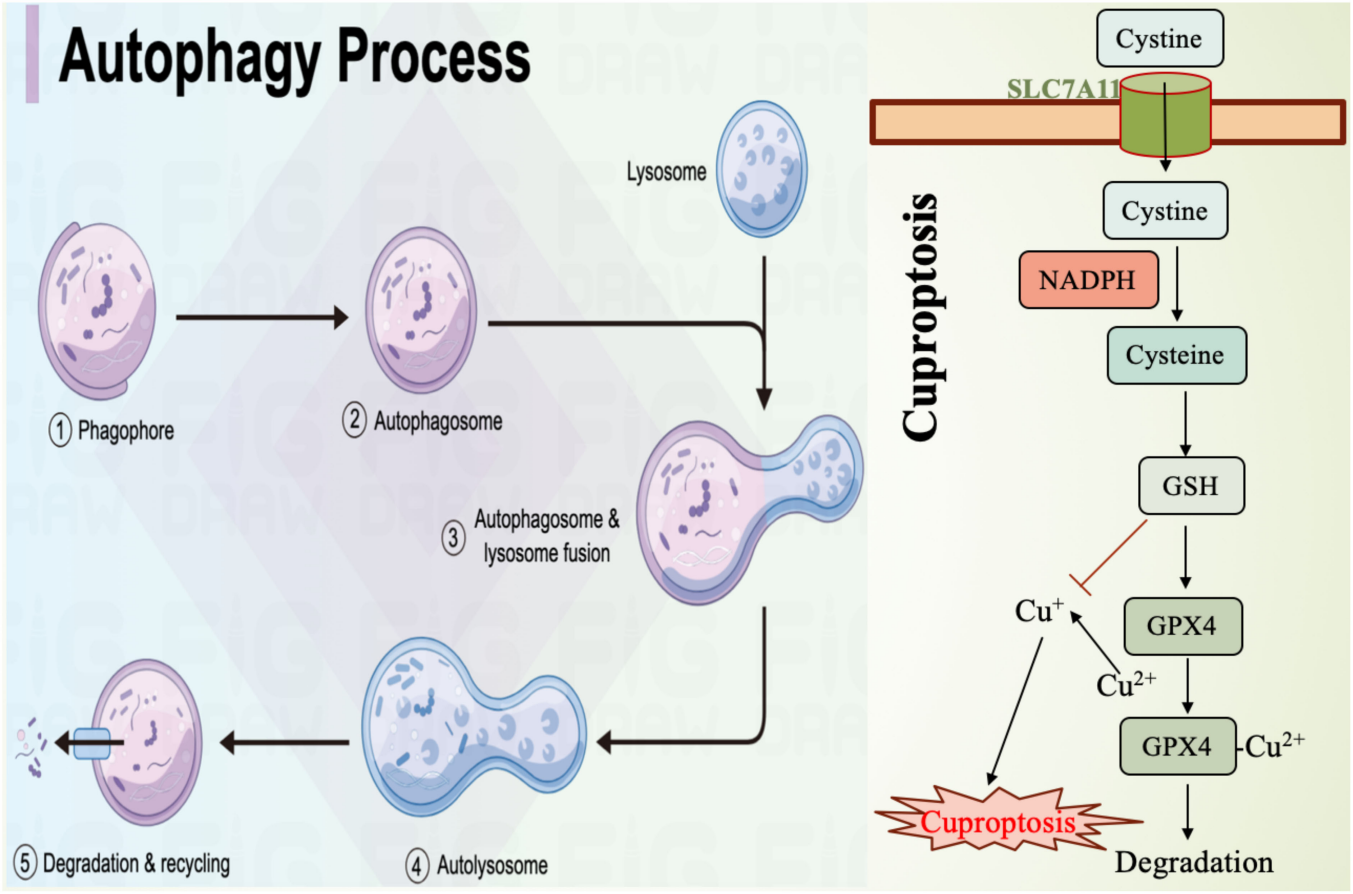
Mechanism diagram of autophagy and cuproptosis. SLC7A11, the cystine transporter solute carrier family member 11; GSH, glutathione; GPX4, glutathione peroxidase 4.

Autophagy is a cellular self-degradation process that clears damaged organelles and proteins, maintaining intracellular homeostasis (**Figure 2**). Recent studies have revealed that autophagy plays a crucial role in promoting ferroptosis. By regulating intracellular iron levels and lipid metabolism, autophagy facilitates the occurrence of ferroptosis. For example, autophagy has been shown to increase the levels of free iron in cells by degrading ferritin, the iron storage protein, thereby accelerating lipid peroxidation and leading to ferroptosis (98). Additionally, oxidative stress is considered a key factor in autophagy-induced ferroptosis. Research indicates that ROS can activate autophagy, further promoting the onset of ferroptosis (99). Therefore, autophagy is not only an important regulatory factor in ferroptosis but also a protective mechanism in cells responding to iron overload and oxidative stress. Inhibition of autophagy significantly impacts ferroptosis. Studies have shown that when autophagy is suppressed, cells become less sensitive to ferroptosis. For example, in cells lacking autophagy-related genes, iron accumulation and lipid peroxidation levels are significantly reduced, thus protecting the cells from ferroptosis (98). Moreover, autophagy inhibitors like 3-methyladenine (3-MA) reduce cell sensitivity to ferroptosis inducers, suggesting that autophagy plays a critical role in regulating ferroptosis (100). In certain pathological conditions, such as atherosclerosis, there is a close interplay between autophagy inhibition and ferroptosis activation, where autophagy suppression alleviates ferroptosis-related cellular damage (101). Therefore, modulating autophagic activity may provide new therapeutic strategies for diseases associated with ferroptosis.

### 5.4 Interaction between ferroptosis and cuproptosis

Cuproptosis is a form of cell death induced by excessive copper, involving mitochondrial dysfunction and protein aggregation (102). Studies have shown that there is a complex interaction between these two cell death mechanisms (**Figure 2**). For example, copper can enhance ferroptosis by promoting the degradation of GPX4 (103). Additionally, copper deficiency increases cellular sensitivity to ferroptosis, suggesting a metabolic crossover between iron and copper. This may occur through the regulation of antioxidant mechanisms and lipid metabolism, thereby influencing each other (104). Understanding this metabolic intersection is crucial for uncovering the regulatory mechanisms of cell death and its role in pathological conditions such as cancer and neurodegenerative diseases. The crosstalk between ferroptosis and cuproptosis involves multiple signaling pathways (**Figure 2**). Research has found that copper accumulation can influence the occurrence of ferroptosis by modulating iron metabolism pathways within mitochondria. This mechanism includes increasing intracellular iron levels by inhibiting enzymes associated with iron metabolism (105). Meanwhile, ferroptosis inducers such as erastin can enhance copper-dependent lipid acylation and protein aggregation, thereby promoting the occurrence of cuproptosis (102). Furthermore, the interaction between iron and copper may also affect cell death fate by regulating intracellular ROS levels. Excessive ROS accumulation causes cellular damage and may promote cell death through the activation of various signaling pathways (106). Therefore, in-depth exploration of the interactions between these signaling pathways could help develop combined therapeutic strategies targeting both ferroptosis and cuproptosis.

As emerging mechanisms of cell death, ferroptosis and cuproptosis show great potential in cancer therapy (**Figure 2**). By targeting and inducing either ferroptosis or cuproptosis, researchers hope to overcome tumor resistance to conventional treatments. For instance, combining ferroptosis inducers with chemotherapy or immunotherapy drugs could significantly enhance anti-tumor efficacy (107). Additionally, copper ion carriers and copper compounds are also being explored as new therapeutic strategies, especially for tumor types that are resistant to standard treatments (108). Moreover, the interaction between ferroptosis and cuproptosis provides new insights for cancer therapy. Studies suggest that targeting both cell death mechanisms simultaneously may yield a synergistic effect, improving treatment outcomes (109). Therefore, a deeper understanding of the mechanisms underlying ferroptosis and cuproptosis and their applications in cancer therapy may offer new treatment options for cancer patients.

The comparison table of ferroptosis and other cell death modes is shown in **Table 2**.

**Table 2:** Comparison of ferroptosis and other cell death modes.

Forms of cell death	Description	Morphological characteristics	Biological significance	Regulatory mechanism	References
Ferroptosis	A type of programmed cell death caused by lipid peroxidation dependent on iron.	Cell membrane rupture, cell shrinkage, mitochondria shrinkage but membrane intact.	Eliminate damaged or abnormal cells to maintain tissue homeostasis.	Regulation of molecules such as GPX4, ACSL4, and FSP1.	(39)
Apoptosis	The process of cell self-destruction controlled by genes is divided into exogenous (death receptor-mediated) and endogenous (mitochondria-mediated) pathways.	The cell membrane remains intact, the cell shrinks, the nucleus fragments, forming apoptotic bodies.	Eliminate excess, damaged, or hazardous cells to maintain tissue homeostasis.	Regulation involving Caspase and the BCL-2 family.	(110)
Autophagy	The process by which cells degrade their own organelles and proteins through the formation of autophagosomes is divided into macroautophagy and microautophagy.	Autophagosome formation encapsulates and degrades organelles and proteins, releasing amino acids and other nutrients for cellular reuse.	Maintain cellular homeostasis to cope with conditions such as hunger and stress.	Regulation involving the ATG family.	(100)
Necroptosis	The way cells suddenly die due to physical, chemical, or biological damage.	Cell membrane rupture, cell swelling, release of cellular contents, leading to an inflammatory response.	The passive process after cell damage may lead to tissue injury and inflammatory response.	Regulation involving mechanisms such as cellular energy depletion and oxidative stress.	(96)
Cuproptosis	The process of programmed cell death caused by lipid peroxidation due to copper ions overload, resulting in the production of a large amount of reactive oxygen species.	Cell volume shrinkage, membrane rupture, organelle swelling, mitochondrial shape changes, while the nucleus remains relatively intact.	It can serve as a potential therapeutic target for anti-tumor treatment by inducing copper death in tumor cells to achieve therapeutic effects.	Regulation by antioxidant enzymes and lipid metabolism enzymes.	(39)

## 6 Therapeutic strategies targeting ferroptosis

### 6.1 Development of ferroptosis inducers

In recent years, the development of ferroptosis inducers has emerged as a novel strategy for cancer treatment. Numerous studies have shown that ferroptosis inducers can effectively induce ferroptosis in tumor cells by targeting iron metabolism and lipid peroxidation, thereby inhibiting tumor growth. In clinical applications, ferroptosis inducers have demonstrated the potential to overcome chemotherapy resistance in certain cancers (111, 112). For example, small molecules such as Erastin, RSL3, and FIN56 have been proven to induce ferroptosis by inhibiting GPX4, offering a new approach for cancer treatment (113, 114).

**Figure 3:**
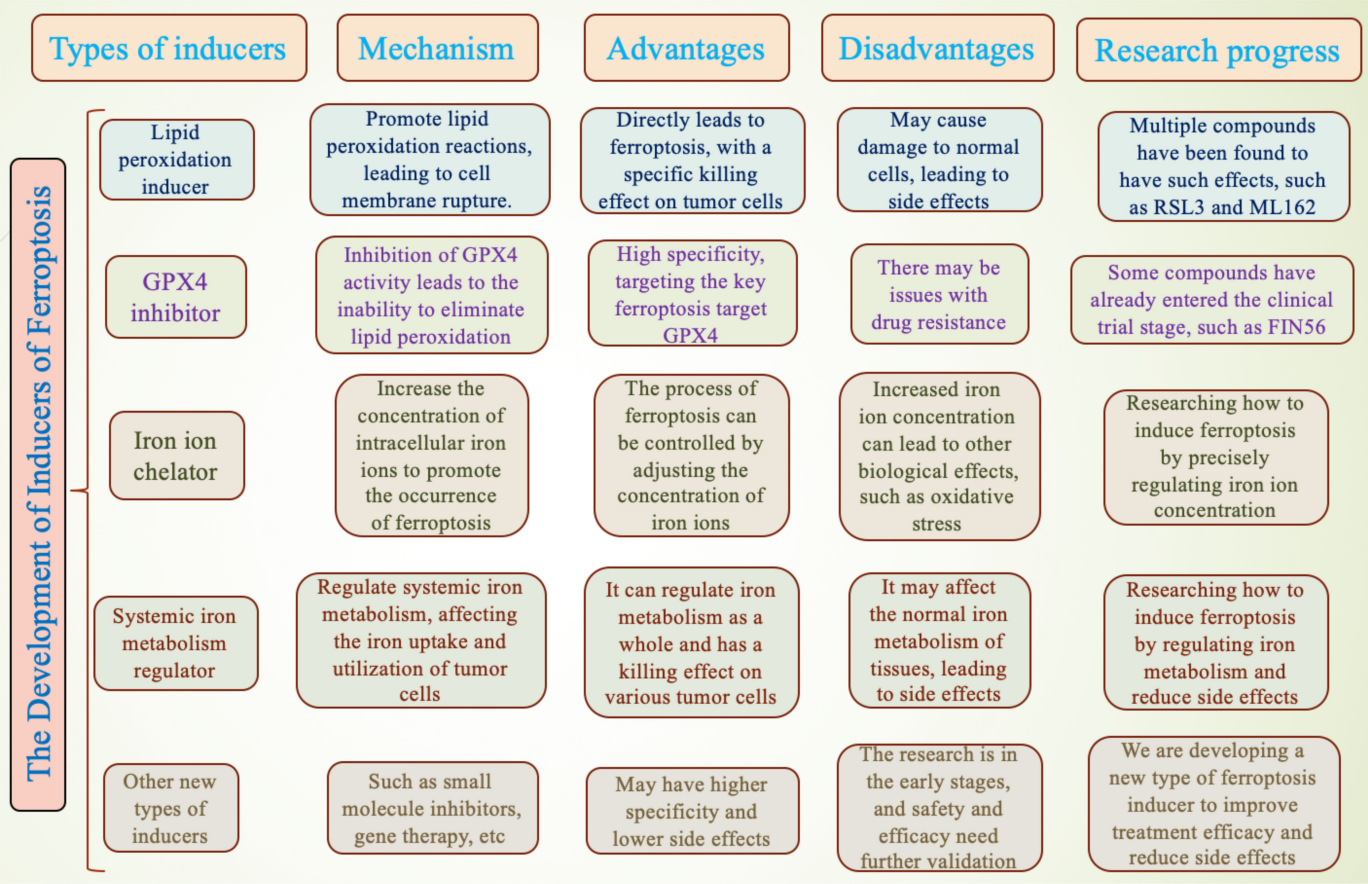
Comparison of various ferroptosis inducers.

Furthermore, advances in nanotechnology have made it possible to target the delivery of ferroptosis inducers, enhancing the bioavailability and therapeutic efficacy of these drugs while reducing toxicity to normal cells (115, 116). Researchers are exploring the use of nanocarriers to improve the specificity and effectiveness of ferroptosis inducers, thus achieving precise targeting of tumor cells (117). Additionally, strategies targeting iron-sulfur clusters (ISCs) have been proposed to induce ferroptosis in cancer cells. By modulating intracellular iron metabolism and lipid peroxidation, ferroptosis inducers are expected to address the issue of resistance in cancer therapies (118, 119). These advances offer new perspectives and possibilities for the clinical application of ferroptosis inducers. A comparison of various ferroptosis inducers is shown in **Figure 3**.

### 6.2 The potential of combined therapy

Combination therapy strategies have shown promising prospects in cancer treatment, particularly when ferroptosis inducers are used in conjunction with other therapeutic modalities. This is because inducing ferroptosis can enhance the sensitivity of tumor cells to other treatments while reducing immune suppression in the tumor microenvironment (120). For example, combining ferroptosis inducers with immunotherapy or targeted therapy has been shown to significantly improve treatment outcomes. Ferroptosis can overcome the resistance of cancer cells to conventional therapies, especially in highly heterogeneous tumors such as glioblastoma and melanoma (121, 122). Studies have found that ferroptosis inducers can enhance the antitumor activity of BRAF inhibitors and immune checkpoint inhibitors, thereby improving the prognosis of patients with malignant melanoma (123).

Radiotherapy combined with ferroptosis inducers has also demonstrated enhanced efficacy, as radiotherapy promotes ferroptosis by increasing intracellular iron accumulation (124). Specifically, radiotherapy induces ROS and activates lipid metabolism enzymes (such as ACSL4), which in turn promote ferroptosis. Moreover, the induction of ferroptosis can enhance the effects of radiotherapy, increasing tumor cell sensitivity and improving treatment outcomes. For instance, certain ferroptosis inducers, when used alongside radiotherapy, significantly enhance the antitumor effects of radiotherapy by increasing the accumulation of lipid peroxides inside cells (125, 126). Clinical studies have shown that combining ferroptosis inducers with radiotherapy can significantly inhibit tumor growth and overcome resistance to radiotherapy in some cancers (127–129).

In the context of chemotherapy, ferroptosis is also considered a novel strategy for overcoming chemotherapy resistance by enhancing the cytotoxic effects of chemotherapeutic agents. Research has shown that combining chemotherapy drugs like cisplatin with ferroptosis inducers can reverse tumor cell resistance, thereby improving the efficacy of chemotherapy (130, 131). This combination strategy not only increases the tumor cell death rate but may also improve patient prognosis, especially in the case of refractory cancers (132, 133). This approach not only overcomes the resistance associated with single therapies but also targets tumor cells through multiple mechanisms, thus enhancing overall treatment efficacy (134).

Studies have also shown that combining therapies with different mechanisms can effectively improve patient response rates and reduce the risk of tumor recurrence (135). However, the specific mechanisms, optimal dosages, and treatment regimens for combination therapy still require further clinical trials and basic research to determine, in order to achieve better therapeutic outcomes and minimize side effects. Therefore, research into the combination of ferroptosis inducers with traditional therapies offers new treatment options and improved prognosis for cancer patients (136).

### 6.3 Preclinical and clinical research status

Currently, therapeutic strategies targeting ferroptosis have made significant progress in preclinical and clinical research. Several studies have validated the antitumor effects of ferroptosis inducers in animal models, demonstrating their potential applications across different cancer types. For example, research on colorectal cancer has shown that ferroptosis inducers can effectively overcome chemotherapy resistance and significantly increase tumor cell death rates (137, 138). In terms of clinical research, although still in its early stages, some small-scale clinical trials are assessing the safety and efficacy of ferroptosis inducers. These studies lay the foundation for future clinical applications and highlight the potential of ferroptosis as a novel therapeutic target (139). Furthermore, these studies not only focus on the direct role of ferroptosis in cancer therapy but also explore its synergistic effects in radiotherapy and immunotherapy (140). While the current findings are promising, more clinical trials are needed to validate the practical effects and indications of ferroptosis-targeted therapy in different cancer types. As our understanding of ferroptosis mechanisms deepens and related drugs continue to be developed, ferroptosis-targeted therapies are expected to become a new option for cancer treatment in the near future.

Research has also explored the impact of modulating ferroptosis-related genes on the prognosis of lung cancer patients, with results indicating that specific ferroptosis-related genes could serve as prognostic biomarkers to predict patient survival. Additionally, clinical trials are exploring strategies to combine ferroptosis inducers with traditional chemotherapy drugs to overcome resistance in lung cancer. Although clinical applications targeting ferroptosis are still in their infancy, the available research results provide a foundation for future clinical trials, suggesting that modulation of ferroptosis could become a new direction for lung cancer treatment.

Ferroptosis, as a novel targeted therapy strategy, demonstrates potential in the treatment of lung cancer. Studies indicate that lung cancer cells are particularly sensitive to ferroptosis, especially in cases of chemotherapy resistance, making ferroptosis induction a promising therapeutic approach (141). For example, certain natural compounds have been found to enhance the effectiveness of chemotherapy for lung cancer by inducing ferroptosis, offering a new solution to chemotherapy resistance. Additionally, researchers are developing new ferroptosis inducers that not only exhibit good selectivity and bioavailability but can also be combined with other treatments such as radiotherapy and immunotherapy to improve therapeutic outcomes. In conclusion, ferroptosis, as a new direction in targeted therapy, holds great promise in the future treatment of lung cancer, particularly in overcoming resistance and enhancing treatment efficacy.

## 7 Conclusion and outlook

In this review, we explored the significance and mechanisms of ferroptosis in lung cancer, emphasizing its role in the tumor microenvironment and its impact on the survival and proliferation of lung cancer cells. Ferroptosis, an iron-dependent form of programmed cell death, has been shown to play a crucial role in various cancer types, particularly in lung cancer. Its importance is highlighted by the complex relationship between ferroptosis and tumor cell survival, proliferation, and drug resistance. Research suggests that ferroptosis not only influences tumor cell survival and proliferation by regulating intracellular iron homeostasis, lipid peroxidation, and antioxidant responses, but it may also alter the sensitivity of tumors to conventional therapies. These findings shed light on the complexity of ferroptosis and its feasibility as a therapeutic target in lung cancer.

Targeting ferroptosis offers a novel perspective for lung cancer treatment. Studies have shown that promoting ferroptosis can effectively suppress tumor cell growth while enhancing responses to chemotherapy and immunotherapy. The potential of this new therapeutic strategy is particularly evident in lung cancer cases resistant to existing treatments. Therefore, the development of drugs or therapies targeting iron metabolism could become a significant direction in lung cancer treatment, warranting further attention from clinical researchers and drug developers. Future research should focus on further elucidating the molecular mechanisms of ferroptosis and its role in the heterogeneity of lung cancer. Additionally, exploring the differences in ferroptosis characteristics between different types of lung cancer (e.g., small cell lung cancer and non-small cell lung cancer) and combining it with other therapeutic approaches will be an important part of future studies. Moreover, understanding the complex interactions between ferroptosis and the tumor microenvironment is crucial for identifying new biomarkers and therapeutic targets.

However, despite the clear potential of ferroptosis in lung cancer treatment, we must be cautious in interpreting existing research results. The factors influencing ferroptosis and the associated signaling pathways vary across different experimental conditions, cell models, and animal studies. There are also some discrepancies in the mechanisms of ferroptosis and its role in lung cancer across different studies. Therefore, it is essential to adopt more standardized experimental designs and conduct multi-center clinical trials to validate the true potential of ferroptosis in lung cancer therapy. Future research should focus on clarifying the molecular mechanisms of ferroptosis and how to effectively leverage this mechanism in clinical practice. Exploring the regulatory factors of ferroptosis in different tumor microenvironments and assessing its combined use with other therapeutic strategies will be key areas of future research. Additionally, developing new ferroptosis inducers and screening biomarkers to predict patients’ responses to ferroptosis-targeted therapies will provide important insights for personalized treatment. Future studies need to be more systematic and in-depth to unravel the complex mechanisms of ferroptosis in the occurrence, progression, and outcome of lung cancer. This will not only enhance our understanding of tumor biology but also lay the foundation for the development of new targeted therapeutic strategies.

Ferroptosis-related therapies hold great potential for clinical application but also face several challenges. Balancing the promotion and inhibition of ferroptosis, ensuring the selectivity and safety of these therapies, is a critical issue that researchers must address. Furthermore, individual differences and the complexity of the tumor microenvironment will affect the clinical efficacy of ferroptosis-based treatments. Therefore, future research should emphasize interdisciplinary collaboration, combining basic research with clinical trials, to facilitate the translation of ferroptosis-related therapies into clinical practice.

In conclusion, ferroptosis, as a novel strategy for lung cancer treatment, shows broad developmental prospects. By integrating multidisciplinary research efforts and delving deeper into the mechanisms of ferroptosis and its potential for clinical application, we hope to provide more effective treatment options for lung cancer patients.
